# Picophotonic localization metrology beyond thermal fluctuations

**DOI:** 10.1038/s41563-023-01543-y

**Published:** 2023-05-11

**Authors:** Tongjun Liu, Cheng-Hung Chi, Jun-Yu Ou, Jie Xu, Eng Aik Chan, Kevin F. MacDonald, Nikolay I. Zheludev

**Affiliations:** 1 Optoelectronics Research Centre and Centre for Photonic Metamaterials, University of Southampton, Southampton, UK; 2 Centre for Disruptive Photonic Technologies, School of Physical and Mathematical Sciences and The Photonics Institute, Nanyang Technological University, Singapore, Singapore; 3 Present Address: School of Physics and Astronomy, University of Southampton, Southampton, UK

**Keywords:** Imaging techniques, Nanophotonics and plasmonics, Nanometrology, Optical metrology, Thermodynamics

## Abstract

Despite recent tremendous progress in optical imaging and metrology^1–6^, there remains a substantial resolution gap between atomic-scale transmission electron microscopy and optical techniques. Is optical imaging and metrology of nanostructures exhibiting Brownian motion possible with such resolution, beyond thermal fluctuations? Here we report on an experiment in which the average position of a nanowire with a thermal oscillation amplitude of ∼150 pm is resolved in single-shot measurements with subatomic precision of 92 pm, using light at a wavelength of *λ* = 488 nm, providing an example of such sub-Brownian metrology with ∼*λ*/5,300 precision. To localize the nanowire, we employ a deep-learning analysis of the scattering of topologically structured light, which is highly sensitive to the nanowire’s position. This non-invasive metrology with absolute errors down to a fraction of the typical size of an atom, opens a range of opportunities to study picometre-scale phenomena with light.

## Abstract (web summary)

The authors report subatomic precision in measuring the displacement of a nanowire. Such precision is achieved by employing deep-learning enabled analysis of single-shot scattering of topologically structured superoscillatory illumination.

## Main

Over the past decade, spatial resolution in far-field optical imaging and metrology has improved far beyond the classical Abbe diffraction limit of *λ*/2, where *λ* is the wavelength of light. A variety of fluorescence- and structured-illumination-based, deterministic and stochastic and single-molecule localization microscopies^1–6^, now commonly used in biological imaging, routinely achieve resolutions of a few tens of nanometres, or better than *λ*/10. The application of artificial intelligence (AI) to the analysis of coherent light scattered by an object offers metrology with an accuracy of only a few nanometres^7^, or better than *λ*/100, on a par with scanning electron microscopy.

Here we demonstrate an approach to optical measurements with a precision level of *λ*/5,300—a length scale equivalent to a fraction of the typical size of an atom, and shorter than the thermal (Brownian) motion amplitude of the target object. In comparison with extreme interferometric techniques, which can provide high sensitivity to changes in the position of a macroscopic object—down to ∼10^−19^ m in gravitational wave detectors with kilometre-scale baselines^8^—our approach allows for single-shot measurements of a micro- to nanoscopic object through a deep-learning-enabled analysis of its scattering pattern when it is illuminated with coherent, topologically structured light containing deeply subwavelength singularity features.

**Fig. 1:**
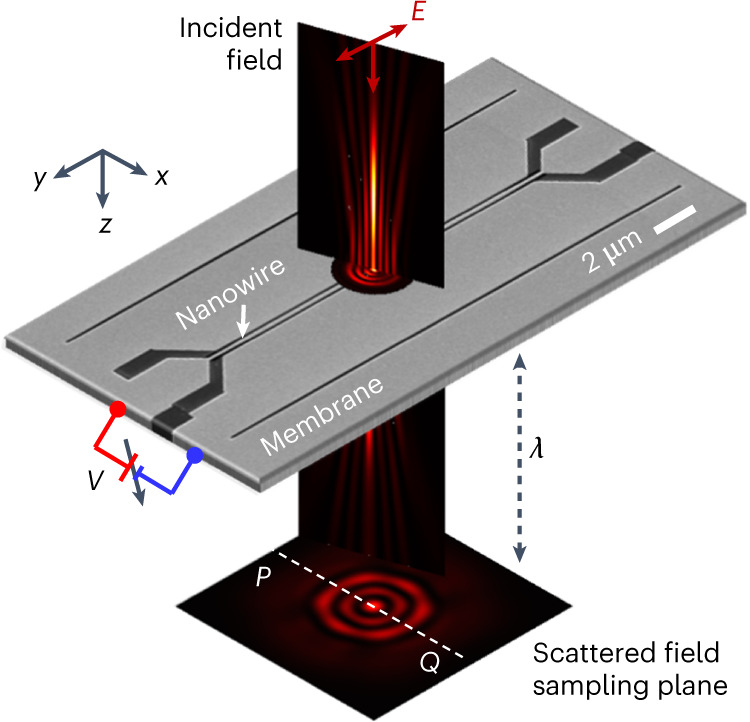
Measuring nanowire displacement via scattering of topologically structured light. Incident light scattered from the nanowire is imaged in transmission through a high-numerical-aperture microscope objective (not shown) focused in a plane located at a distance *λ* behind the membrane. Deeply subwavelength lateral (*x* direction) displacements of the wire, controlled by application of a d.c. bias between the wire and the adjacent edge of the supporting membrane, are quantified via a deep-learning-enabled analysis of single-shot scattering patterns.

In our experiment, we measured the in-plane position of a suspended 17-μm-long, 200-nm-wide nanowire, cut by focused ion beam milling from a 50 nm thick Si_3_N_4_ membrane coated with a 65 nm layer of gold, relative to the fixed edges of the surrounding membrane ~100 nm away on either side (Fig. 1). This position—that is, the displacement of the nanowire from the mid-point between two edges of the slit—can be controlled electrostatically with high accuracy over a few-nanometre range through the application of a d.c. bias across the gap. The sample was illuminated by coherent light at a wavelength of *λ* = 488 nm, with either a plane (defocused Gaussian) wavefront or a superoscillatory wavefront profile formed by a spatial light modulator-based wavefront synthesizer (see Supplementary Information, section 1). The intensity pattern of light scattered by the nanostructure was imaged in transmission in a sampling plane at a distance of ~*λ* (∼0.5 μm) from the membrane by a 16-bit image sensor through a microscope objective with a numerical aperture of 0.9.

To enable optical measurements of unknown nanowire positions via deep learning, we created a dataset of single-shot (401 pixel × 401 pixel) scattering patterns recorded in random sequence at 301 different (electrostatically controlled) positions of the nanowire, up to a maximum displacement of ~4,500 pm. To exclude any effect of stress history in the nanowire, its position was reset to zero between each measured position. Knowledge of the nanowire position as a function of applied bias was obtained from independent measurements using a scanning electron microscope. Eighty percent of the images (selected at random), and the corresponding nanowire positions, were used to train a neural network to enable subsequent retrieval (by the trained network) of unspecified nanowire positions from previously unseen single-shot scattering patterns (that is, the other 20% of the dataset). Details of the network architecture and training procedure, and positional calibration measurements are given in Supplementary Information, section 2. This exercise was conducted for regimes of both plane-wave and superoscillatory illumination, and repeated 20 times using different random selections of training and testing images each time (Supplementary Information, section 2).

**Fig. 2:**
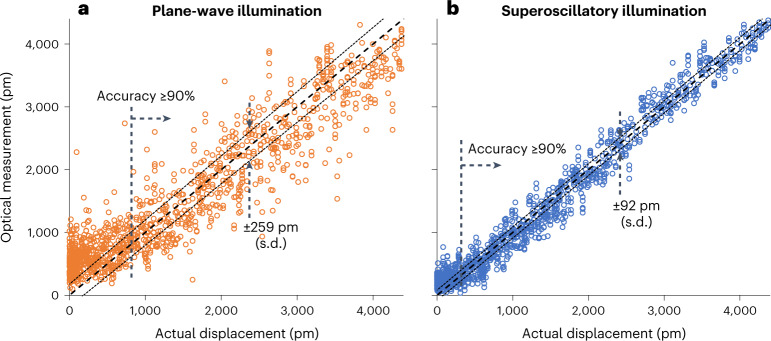
Optical measurements of nanowire displacement. **a**,**b**, Optically measured versus actual values of nanowire displacement for plane-wave (**a**) and topologically structured superoscillatory (**b**) illumination. Dotted lines above and below the ideal correlation diagonals are plotted at ±1 s.d. Dashed vertical lines denote minimum nanowire displacements that can be measured with accuracy exceeding 90% (relative error, <10%).

Figure 2 shows optically measured nanowire positions, retrieved by the trained neural network from scattering patterns, against actual nanowire positions, independently evaluated from electron microscope measurements, for plane-wave (Fig. 2a) and superoscillatory (Fig. 2b) illumination. The statistical spread of data points is derived from the 20 independent neural network training and testing cycles. We characterize the performance of the optical technique in terms of precision—a figure of merit for how close repeated measurements at a given value of actual displacement are to one another (evaluated in terms of measurement standard deviation (s.d.), averaged over the entire range of measured values)—and accuracy—how close measurements are to the true value (evaluated in terms of relative error; that is, the difference between measured and true values as a percentage of the true value).

Our results show that nanowire displacements can be measured with a precision (Fig. 2b) of 92 pm with superoscillatory illumination, and 259 pm using plane-wave illumination. At a wavelength of 488 nm, a precision of 92 pm represents a level of *λ*/5,300, and should be compared with the 775 pm lattice parameter of Si_3_N_4_ (the material from which the nanowires are manufactured) and the ~220 pm covalent diameter of a silicon atom. With superoscillatory illumination, accurate measurements are possible for displacements down to a few hundred picometres, with relative error ≤10% down to 320 pm; for plane-wave illumination, this accuracy threshold is 816 pm.

Optical metrology based up analysis of scattered light is an inverse problem that can be reduced to the Fredholm integral equation, which can be efficiently solved by a neural network. Indeed, recent work has demonstrated that this approach yields accuracy better than *λ*/100 in measuring the width of gaps in an opaque film with plane-wave illumination, using a neural network trained on a set of nanofabricated samples with a range of different gap sizes^7^. There are two major contributing factors to the hundred-fold improvement in precision reported here: a markedly better training process and the use of topologically structured superoscillatory light.

Precisely tailored interference of multiple waves can form intensity ‘hotspots’ in free space, with dimensions smaller than the conventional diffraction limit, as a manifestation of what is known as superoscillation^9^. The electromagnetic field near a superoscillatory hotspot has many features similar to those in the vicinity of resonant plasmonic nanoparticles or nanoholes—hotspots are surrounded by phase singularities and nanoscale zones of energy backflow where phase gradients can be orders of magnitude larger than in a free propagating plane waves^10^. The use of such topologically structured light gives an advantage for AI-enabled metrology: the ability to evaluate small changes in the position of the nanowire depends upon the magnitude of associated changes in the scattered light field at distance *z* from the object $A\left( {x,z} \right)e^{i\phi \left( x \right)} = f\left( {A_0\left( {x,0} \right)e^{i\phi _0\left( {x,0} \right)}} \right)$, where *A*_0_(*x*,0) is the amplitude and *ϕ*_0_(*x*, 0) is the phase of the incident light in the *xy* object plane. A small displacement of the object against the incident field in the *x* direction results in a change in scattered light intensity $\delta I\left( x \right)\approx \delta A_0\left( {x,0} \right)^2 + A_0\left( {x,0} \right)^2\delta \phi _0\left( {x,0} \right)^2$. The first term on the right-hand side is related to the change of illumination intensity associated with the object’s positional shift, while the second relates to the corresponding change in phase. The phase-dependent term is absent for plane-wave illumination, but can be large under superoscillatory illumination, when the object traverses a small (deeply subwavelength) feature of the incident field, such as singularity, where the phase *ϕ*_0_(*x*,0) jumps by π.

**Fig. 3:**
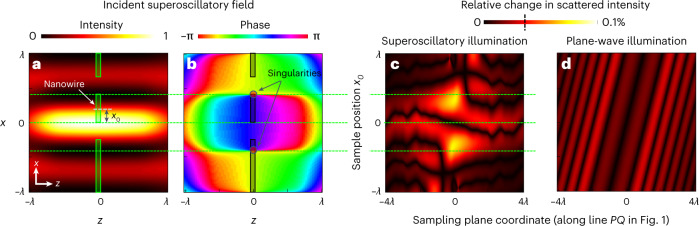
Sensitivity of scattered fields to small nanowire displacements. **a**,**b**, Intensity (**a**) and phase profiles (**b**) of the superoscillatory field in the *xz* plane (light propagating in the +*z* direction, wavelength *λ* = 488 nm). The sample—a nanowire in the gap between two semi-infinite sections of membrane—lies in the *z* = 0 plane (its cross-sectional profile being shown in green in **a** and grey in **b**). **c**, Relative change in scattered light intensity resulting from a *λ*/1,000 displacement of the sample in the *x* direction along a cross-sectional line through the scattering pattern in the sampling plane (line PQ in Fig. 1) as a function of the initial position *x*_0_ of the sample relative to the symmetry axis of the light field. **d**, Corresponding plot of relative change in scattered light intensity for plane-wave illumination of the same sample structure. (Further details of numerical simulations are given in Supplementary Information, section 3).

The responses of scattered plane-wave and topologically structured light fields to displacement of an illuminated nanowire are illustrated, through computational modelling, in Fig. 3. The incident superoscillatory wavefront (detailed in ref. ^10^) has a central intensity maximum (Fig. 3a) flanked by phase singularities and zones of high phase gradient (Fig. 3b). We consider the case here where these singularities lie in the nanowire sample plane. As a figure of merit for the sensitivity of the scattered field to small displacements of the nanowire, Fig. 3c presents the magnitude of the relative change in scattered light intensity induced by a *λ*/1,000 (~0.5 nm) shift in nanowire position as a function of (horizontally) the image plane coordinate and (vertically) the initial position of the sample within the structured light field. The scattered field intensity is strongly dependent on both, with the largest changes (of up to 0.1%) occurring when a sharp edge of the nanostructure coincides with a phase singularity in the incident superoscillatory field. For comparison, Fig. 3d shows the same for plane-wave illumination. Here, the variations in scattered field intensity are smaller (reaching only 0.03%) and relatively weakly dependent on both image plane coordinate and lateral position of the sample. The contrast between Fig. 3c and d explains the better precision of positional measurement achieved with superoscillatory, as compared to plane-wave, illumination (Fig. 2).

The quality of AI is directly related to the quality of training data for the neural network. Our ability here to achieve picometric levels of precision results primarily from the use of a training dataset that is ultimately congruent with the object of interest: the same electrostatically reconfigurable nanostructure is used for training and as the object of metrological study. Moreover, ground truth positional displacement values are more precisely calibrated in this singular, electrostatically controlled gap sample (Supplementary Fig. 1) than they are in previously employed sets of discrete training samples fabricated with typically few-nanometre tolerance^7^. It should further be noted that access to this low level of measurement error is only possible because the deep-learning-based retrieval process is sensitive primarily to the intensity profile of the diffraction pattern, but less so to its lateral position. As such it can be strongly resilient to small thermal and mechanical drifts in the mutual positions of the lens, sample and incident light beam during the experiment^11^.

Our results represent the first example of sub-Brownian optical metrology—the measurement of object dimensions/displacements with precisions smaller than the amplitude of its thermal motion. The amplitude of nanowire thermal vibration can be evaluated from the Langevin oscillator model^12^: in the present case, the nanowire’s fundamental in-plane and out-of-plane oscillatory modes, at 1.6 and 1.1 MHz, respectively, have amplitudes of 145 and 215 pm at room temperature (Supplementary Information, section 4), that is, values larger than the achieved superoscillatory illumination measurement precision of 92 pm. Our measurements are performed with a detector integration time of ~100 ms and thus return the mean position of the nanowire, which oscillates thermally with a much shorter (~0.6 μs) period. However, the measurements are single-shot and do not require scanning of the object, and therefore they can be performed with a frame rate equal to that of the image sensor, which may reach tens of megahertz in advanced ultrafast cameras. It should be noted here that the effect of optical forces on nanowire position is negligible (Supplementary Information, section 5).

Localization precision surpassing the diffraction limit of conventional microscopes thousands of times over has been demonstrated here on a system allowing for the collection of in situ physical training data for the neural network. Although a dependence on in situ training may be seen as a limitation, the approach can be applied in a variety of systems where a regime of externally controlled positioning is available for training, to then enable non-invasive study of motion induced by ambient forces and fields (internal or external to the object), or thermal motion. This may be, for example in: non-contact position monitoring of platforms in ultraprecise scanning tunnelling and atomic force microscopes; monitoring positional displacements in micro- and nanoelectromechanical systems devices such as accelerometers and nanomachines; monitoring structural deformations and thermal drifts in precise instruments (for example, telescope mirrors) and seismographs; and measuring the thermal expansions of macroscopic objects and monitoring nanogaps affected by microkelvin temperature variations. Moreover, there is growing interest in short-range forces and associated phenomena at the nano- to picoscale, which may be investigated via comparative studies in in situ trained systems designed to enhance or suppress the mechanism of interest, or where external stimuli (for example, light) can be selectively applied or withdrawn. Forces of interest may include, for example: ponderomotive optical, Van der Waals, Casimir, and recently identified non-Hamiltonian and spin-related optical forces^13,14^, which manifest at nano- to picometre scales. Indeed, while our technique cannot compete with Laser Interferometer Gravitational-Wave Observatory (LIGO)-type macroscopic platform displacement measurements on kilometric baselines for gravitational wave detection^8^, it may be useful in the study of microgravitational forces on micro/nano-objects at very short distances^15^. With ultrafast image sensors (>10 Mfps, as are now becoming accessible), our method may be applied to the study of dynamics and transient processes at the (sub)nanoscale. For example, the study of Brownian motion thermodynamics of nano-objects, including the ballistic regime^16^ and non-Markovian processes of thermal fluctuation, which may have applications in fast thermometry and mass measurement; the study of electron and plasmon quantum transport through atomic-scale gaps^17–19^; configuration chemistry; protein folding^20^; and other time-dependent events in macromolecules, nanomachines and two-dimensional materials, where flexural, phononic modes are now understood to be of critical importance to thermal, electrical and mechanical properties^21–23^.

Optimization of the incident field topology, beyond the superoscillatory wavefront used in this study, is expected to allow for even higher measurement precision in comparison to plane-wave illumination. As for any other optical visualization technique, performance of the reported method depends upon the refractive index contrast between the object of interest and its surroundings and, as such, it may be efficiently applied to localization studies in metallic, high-index dielectric and semiconductor nanostructures and metamaterials.

In conclusion, this work opens up the exciting field of picophotonics—the study of light–matter interactions and phenomena at the atomic scale.

## Online content

Any methods, additional references, Nature Portfolio reporting summaries, source data, extended data, supplementary information, acknowledgements, peer review information; details of author contributions and competing interests; and statements of data and code availability are available at 10.1038/s41563-023-01543-y.

### Supplementary information

Supplementary Information Supplementary discussion sections 1–5 and Fig. 1.

## Data Availability

For the purpose of open access, the authors have applied a Creative Commons attribution (CC BY) license to any author accepted manuscript version arising. The data from this paper can be obtained from the University of Southampton ePrints research repository: 10.5258/SOTON/D2544.
