# Supplementary material for: Picophotonic localization metrology beyond thermal fluctuations
Source: Nat Mater. 2023 May 11;22(7):844–7. doi: 10.1038/s41563-023-01543-y (PMC10691967; doi:10.1038/s41563-023-01543-y)
Supplement: Supplementary file 1 — Supplementary discussion sections 1–5 and Fig. 1. [file 41563_2023_1543_MOESM1_ESM.pdf]

nature materials

Letter https://doi.org/10.1038/s41563-023-01543-y

Picophotonic localization metrology beyond thermal fluctuations

In the format provided by the authors and unedited

Contents

S1 – Wavefront synthesis

S2 – Neural network architecture, training, and application procedures

S3 – Numerical modelling of light scattering

S4 – Thermal fluctuations of the nanowire

S5 – Optical forces acting on the nanowire

S1: Wavefront synthesis

The computer-controlled wavefront synthesizer employed in this work is described in detail in Ref. 1. It is based upon a pair of (Meadowlark P512) spatial light modulators – one for intensity and the other for phase modulation.

For the purpose of this study we employ an axially-symmetric superoscillatory wavefront constructed from just two circular prolate spheroidal wavefunctions, *S3* and *S4* (following Rogers, *et al.*[2]): $E(r/\lambda)$ = 4.477 $S3(r/\lambda)$ + $S4(r/\lambda)$, where r is radial distance from the beam axis. This simple analytical form considerably simplifies optimization of the experimental wavefront synthesizer, as only one free (relative weighting) parameter is involved.

In the 'plane wave' illumination regime, the synthesizer was configured to generate a defocused Gaussian beam profile having a (measured) intensity variance of only ±5% over the ~400 nm width of the sample (i.e. including the nanowire and gap on either side).

S2: Neural network architecture, training, and application procedures

The neural network contained three convolution layers with, respectively, sixty-four 5×5, one hundred and twenty-eight 4×4, and two hundred and fifty-six 2×2 kernels, and three fully connected layers with 128, 256, 128 neurons. Each of the convolution layers was followed by a pooling layer with 4×4, 3×3, and 3×3 kernels with Rectified Linear Unit activation functions. The network was trained with the Adam stochastic optimization method and root mean square error loss function. The network was optimized by searching the hyperparameter space (adjusting the number of layers, number of neurons in each layer, and their activation functions) to simultaneously minimize training time and validation loss during training.

Our datasets comprise intensity patterns of transmitted light scattered by the nanowire at different electrostatically-controlled in-plane positions relative to the edges of the gap in the membrane. The patterns were imaged within a $10.3\lambda \times 10.3\lambda$ (401 × 401 pixels) field of view of at a distance λ from the sample. The scattering patterns were recorded at 301 different positions of the nanowire over a range from 0 to ~4.4 nm (applied bias settings of 0-2.1 V at intervals of 7 mV) in random sequence to excludes the possibility of neural network learning based upon any artefacts in the patterns other than those associated with changes in nanowire position. To eliminate any effect of stress history in the nanowire, its position was also reset to zero between each recorded position.

64% of scattering patterns (selected at random) were used for network training and 16% for validation, with the remaining 20% then employed for testing (i.e. as scattering patterns for nominally unknown nanowire positions, to be determined by the trained network). To exclude any dependence of measurement outcome on the selection of training scattering patterns and their order of appearance in the training process, twenty independent iterations of the training, validation and testing procedure were performed for each regime of illumination.

The recording of a complete set of 301 network training, validation and test images takes approximately 4 minutes. Over such a period, instrumental alignment fluctuations due to ambient mechanical noise and thermal instabilities (e.g. in the microscope frame, sample stage, etc.) may be orders of magnitude larger than the precision achieved in optical localization of the nanowire position relative to the slit edges. This is a strong indication that neural network training results in a retrieval algorithm which principally recognizes the structure of the scattering pattern created by the nanowire in the gap rather than its position on image sensor.

Ground truth values of nanowire displacement were independently established by *a priori* measurements under a scanning electron microscope for a number of different bias settings and interpolated by a quadratic dependence (Fig. S1): the first non-zero term in the analytical expression for the dependence of nanowire displacement (*D*) on applied bias (*V*) must be quadratic as displacement does not depend on the sign of the bias; and higher order terms are negligible while the magnitude of displacement remains much smaller that the gap size (of ~100 nm). Although each individual measurement by scanning electron microscope has an uncertainty of order ±1 nm (related to SEM image pixelation), measurements over a range of applied bias values enables accurate determination of the quadratic dependence $D = \alpha V^2$, where α takes a value of 1.0328 nm/V^2 with a standard error of 0.0053, in the present case. The resulting uncertainty in absolute calibration of displacement (applicable to both the actual and optically measured scales), at ~1.4%, is smaller than the achieved precision of optical measurements over the full measurement range.

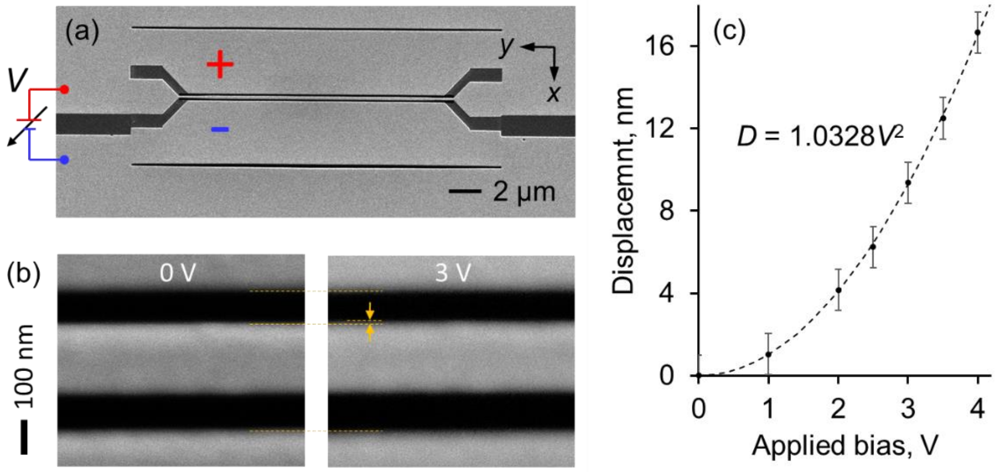

Fig. S1. Nanowire position calibration. (a) SEM image of the entire nanowire sample, showing the electrode configuration for electrostatic control of [*x* direction] position; (b) representative pair of high magnification images of the (*y* direction) midpoint of the nanowire from which the dependence of nanowire displacement on applied bias – panel (c) – is derived. [Error bars in (c) denote the uncertainty associated with a ±1 pixel error in determining the nanowire edge position from SEM images.]

S3: Numerical modelling of light scattering

Numerical simulations pertaining to the sensitivity of light scattering to small nanowire displacements (Fig. 3 in the manuscript) were performed using Lumerical FDTD Solutions. Silicon nitride is taken to have a refractive index n = (2 + 0i), while parameters for gold are those by Johnson & Christy. Incident light is polarized parallel to the nanowire and perfectly matching layer (PML) boundary conditions are used. The incident superoscillatory field was generated through a binary amplitude mask as detailed in Ref. 3.

S4: Thermal fluctuations of the nanowire

The thermal motion of nanomechanical structures is described by the Langevin model[4]. For a harmonic oscillator

$$\ddot{x} + \gamma\dot{x} + \omega_0^2 x = F_T(t)/m_{eff}$$

where

- $F_T(t) = \sqrt{2k_B T\gamma/m_{eff}}\eta(t)$ is the thermal force [which is related to the dissipation factor γ through the fluctuation-dissipation theorem[5]];
- k_B is the Boltzmann constant;
- T is temperature;
- $\eta(t)$ is a delta-correlated normalized white noise term: $\langle\eta(t)\rangle = 0$; $\langle\eta(t)\eta(t')\rangle = \delta(t - t')$;
- $\omega_0 = 2\pi f_0 = \sqrt{(k/m_{\text{eff}})}$ is the natural angular frequency of oscillation, f_0 being the natural frequency and k the spring constant;
- and m_{eff} is the oscillator's effective mass;

the RMS beam displacement is

$$\delta x_{\text{RMS}} = \sqrt{k_B T/(4\pi^2 m_{\text{eff}} f_0^2)}$$

In the present case, m_{eff} = 2 pg and for the in- and out-of-plane modes of oscillation respectively f_0 = 1.6 and 1.1 MHz, giving an average thermal fluctuation amplitudes of ~145 and ~215 pm.

S5: Optical forces acting on the nanowire

From numerical modelling of ponderomotive and radiation pressure forces, we conclude that within accuracy of the experiment they are insignificant to corrupt the ground truth values for the nanowire position.

To evaluate the action of these optical forces on the nanowire, we evaluated the Maxwell stress tensor in FDTD numerical simulations (Lumerical). We consider plane wave and superoscillatory incident wavefronts close to those used in experiment, with a total incident power of 100 µW over an 8 µm × 8 µm area of the sample. We then evaluate displacements induced by these forces, assuming a nanowire spring constant $k = m_{eff}\omega_0^2$ = 0.2 Nm^{-1} (see section S4 above). In both illumination regimes, the optically-induced in-plane (*x* direction) displacement is zero when both the nanowire and incident light field are centered on the gap in the membrane, and it grows to not more than a few femtometers when the nanowire is laterally displaced (i.e. in experiment, electrostatically) by up to 5 nm. This represents a negligible perturbation against the tens of picometers best accuracy achieved in optical measurements of said displacement.

In the *z* direction perpendicular to the sample plane, optically-induced nanowire displacement (due to radiation pressure) may reach several tens of femtometers - still a very small amount. Moreover, the membrane on either side of the nanowire will be subject to comparable forces, whereby relative *z* displacement between nanowire and membrane will be much smaller, if not near-zero.

References

1 Rogers, E. T. F. *et al.* Far-field unlabeled super-resolution imaging with superoscillatory illumination. *APL Photon.* **5**, 066107 (2020).

2 Rogers, K. S., Bourdakos, K. N., Yuan, G. H., Mahajan, S. & Rogers, E. T. F. Optimising superoscillatory spots for far-field super-resolution imaging. *Opt. Express* **26**, 8095-8112 (2018).

3 Yuan, G., Rogers, E. T. F. & Zheludev, N. I. "Plasmonics" in free space: observation of giant wavevectors, vortices, and energy backflow in superoscillatory optical fields. *Light Sci. Appl.* **8**, 2 (2019).

4 Wang, M. C. & Uhlenbeck, G. E. On the Theory of the Brownian Motion II. *Rev. Mod. Phys.* **17**, 323-342 (1945).

5 Kubo, R., Toda, M. & Hashitsume, N. *Statistical Physics II: Nonequilibrium Statistical Mechanics*. Vol. 31 (Springer, 1991).
